# A coral-associated actinobacterium mitigates coral bleaching under heat stress

**DOI:** 10.1186/s40793-023-00540-7

**Published:** 2023-11-23

**Authors:** Jie Li, Yiyang Zou, Qiqi Li, Jian Zhang, David G. Bourne, Yuanjiao Lyu, Cong Liu, Si Zhang

**Affiliations:** 1 CAS Key Laboratory of Tropical Marine Bio-resources and Ecology, South China Sea Institute of Oceanology, Chinese Academy of Sciences, Guangzhou, Guangdong China; 2 Sanya National Marine Ecosystem Research Station, Chinese Academy of Sciences, Sanya, Hainan China; 3 College of Science and Engineering, James Cook University, Townsville, QLD Australia; 4 Australian Institute of Marine Science, Townsville, QLD Australia

**Keywords:** Thermal stress, Coral bleaching, An actinobacterial probiotic, Coral microbiome, Ecological network, Coral holobiont, Symbiodiniaceae, Transcriptome, Nutrient acquisition

## Abstract

**Background:**

The positive effects of exposing corals to microorganisms have been reported though how the benefits are conferred are poorly understood. Here, we isolated an actinobacterial strain (SCSIO 13291) from *Pocillopora damicornis* with capabilities to synthesize antioxidants, vitamins, and antibacterial and antiviral compounds supported with phenotypic and/or genomic evidence. Strain SCSIO 13291 was labeled with 5 (and − 6)-carboxytetramethylrhodamine, succinimidyl ester and the labeled cell suspension directly inoculated onto the coral polyp tissues when nubbins were under thermal stress in a mesocosm experiment. We then visualized the labelled bacterial cells and analyzed the coral physiological, transcriptome and microbiome to elucidate the effect this strain conferred on the coral holobiont under thermal stress.

**Results:**

Subsequent microscopic observations confirmed the presence of the bacterium attached to the coral polyps. Addition of the SCSIO 13291 strain reduced signs of bleaching in the corals subjected to heat stress. At the same time, alterations in gene expression, which were involved in reactive oxygen species and light damage mitigation, attenuated apoptosis and exocytosis in addition to metabolite utilization, were observed in the coral host and Symbiodiniaceae populations. In addition, the coral associated bacterial community altered with a more stable ecological network for samples inoculated with the bacterial strain.

**Conclusions:**

Our results provide insights into the benefits of a putative actinobacterial probiotic strain that mitigate coral bleaching signs. This study suggests that the inoculation of bacteria can potentially directly benefit the coral holobiont through conferring metabolic activities or through indirect mechanisms of suppling additional nutrient sources.

**Supplementary Information:**

The online version contains supplementary material available at 10.1186/s40793-023-00540-7.

## Introduction

Coral reefs are the largest structures created by living organisms and are home to highly diverse marine organisms [1]. Reef-building corals are associated with a diverse microbiota embracing photosynthetic Symbiodiniaceae, as well as bacteria, fungi, archaea, and viruses, which together with their coral host comprise the holobiont [2]. Symbiodiniaceae fix carbon and provide photosynthates to the coral host. Other microbial symbionts also play crucial roles in maintaining coral host fitness and survival by participating in carbon, nitrogen, and sulfur cycling, providing nutrients, and defending against pathogens [2].

The coral-associated microbiome is sensitive to environmental perturbation [2]. The coral-Symbiodiniaceae symbiosis may be disrupted by thermal stress, resulting in the elimination of algal endosymbionts and coral bleaching, leading to a shortage of energy for coral hosts [3]. It is known that elevated temperature damages Symbiodiniaceae’s photosynthetic apparatus and leads to the generation of reactive oxygen species [3]. Oxidative stress due to increased temperatures can activate innate immunity in corals, and host cells undergoing apoptosis seek to remove damaged symbionts by host cell suicide [3, 4]. Exocytosis of algal symbionts has also been observed in coral bleaching [3]. In addition to oxidative stress induced by elevated temperature, nutrient reduction due to damaged photosystems in Symbiodiniaceae may also lead to coral bleaching through the photooxidative pathway [5]. At the molecular level, heat shock proteins and antioxidant enzymes are commonly found to be upregulated in corals in response to heat stress [3, 4, 6–8]. The overexpression of *p*53, a pro-apoptotic transcription factor, after exposure to thermal stress activates caspases, which are involved in apoptosis and increase under thermal stress in corals [7–10]. Symbiodiniaceae also exhibited significant expression alterations in the face of elevated temperatures, such as genes associated with photosynthesis, metabolism, antioxidant activity, and the immune response [7, 8, 11–13].

Globally, coral reefs are in decline largely as a result of increased scale, frequency, and intensity of coral bleaching events driven by anthropogenic global warming [14–16]. Given the significant functional roles of the bacterial symbionts in the coral holobiont [2, 17], the application of beneficial microorganisms for corals (BMCs) to enhance coral tolerance and resilience to environmental stress is promising and has received increasing attention in recent years [18, 19]. By selecting bacterial isolates with potential beneficial traits to corals, such as facilitating enhanced nitrogen (e.g. fixation and denitrification) or sulphur cycling, catalase activity, and inhibition activity to putative pathogens (e.g. *Vibrio coralliilyticus*), assembled BMC consortiums applied to corals have been shown to mitigate coral bleaching and mortality when corals were challenged with higher seawater temperatures or putative coral pathogens [18–20]. Currently however, it is not clear if the added microbial strains successfully establish a symbiotic relationship with the corals and confer microbial mediated traits that benefit the host or indirectly buffer coral stress through provision of additional nutrients [18–21].

Actinobacteria are ubiquitously associated with stony corals [22, 23], with several culturable coral-associated actinobacteria demonstrating high antimicrobial activity and the potential to synthesize a range of putative beneficial biosynthetic compounds [24]. However, the probiotic potential of actinobacterial strains has not been investigated extensively [19]. During the exploration of culturable coral-associated bacteria, we isolated the facultative anaerobic Actinobacteria strain SCSIO 13291 from *Pocillopora damicornis* tissues. Here, we explored the genetic and phenotypic properties of this strain. Given its capabilities, we further tested if this strain was capable of ameliorating environmental stress when inoculated onto the coral *P. damicornis* exposed to controlled thermal stress. Bacterial cells were labelled with fluorescent dye and inoculated onto the tissues of coral polyps with visualization, coral physiological, transcriptome and microbiome analyses conducted to elucidate the interactions and effect this strain conferred on the coral holobiont.

## Results

### Genetic and phenotypic characteristics of strain SCSIO 13291

Phylogenetic analysis of the complete 16S rRNA gene sequence of strain SCSIO 13291 indicated it was a member of the family Propionibacteriaceae (Figure S1) and shared the highest similarity (99.87%) to *Propioniciclava soli* YIM S02567^T^ described recently [25]. The average nucleotide identity (ANI) and digital DNA–DNA hybridization (dDDH) values between genomes of SCSIO 13291 and *P. soli* YIM S02567^T^ were 98.71% and 86.2%, respectively, supporting the identification of strain SCSIO 13291 as a *P. soli* species.

Colonies of strain SCSIO 13291 were facultative anaerobic and catalase positive. Extracellular yields of folic acid and vitamin B_3_ from strain SCSIO 13291 were 35.71 ± 6.64 µg L^− 1^ and 5.27 ± 0.56 mg L^− 1^, respectively. The strain also had the capacity for heme production (8.46 ± 1.43 µg g^− 1^ wet weight) while also possessing the complete biosynthesis pathway. Several secondary metabolite biosynthetic gene clusters were also found in the genome including two terpene clusters, one non-ribosomal peptide synthetase cluster, one Class III lanthipeptide cluster like labyrinthopeptin, and one Class I lanthipeptide cluster like nisin. Other morphological, physiological, biochemical and genome properties of strain SCSIO 13291 are described in the supplementary material (Dataset S1).

### Inoculation of strain SCSIO 13291 mitigated coral bleaching signs

**Fig. 1:**
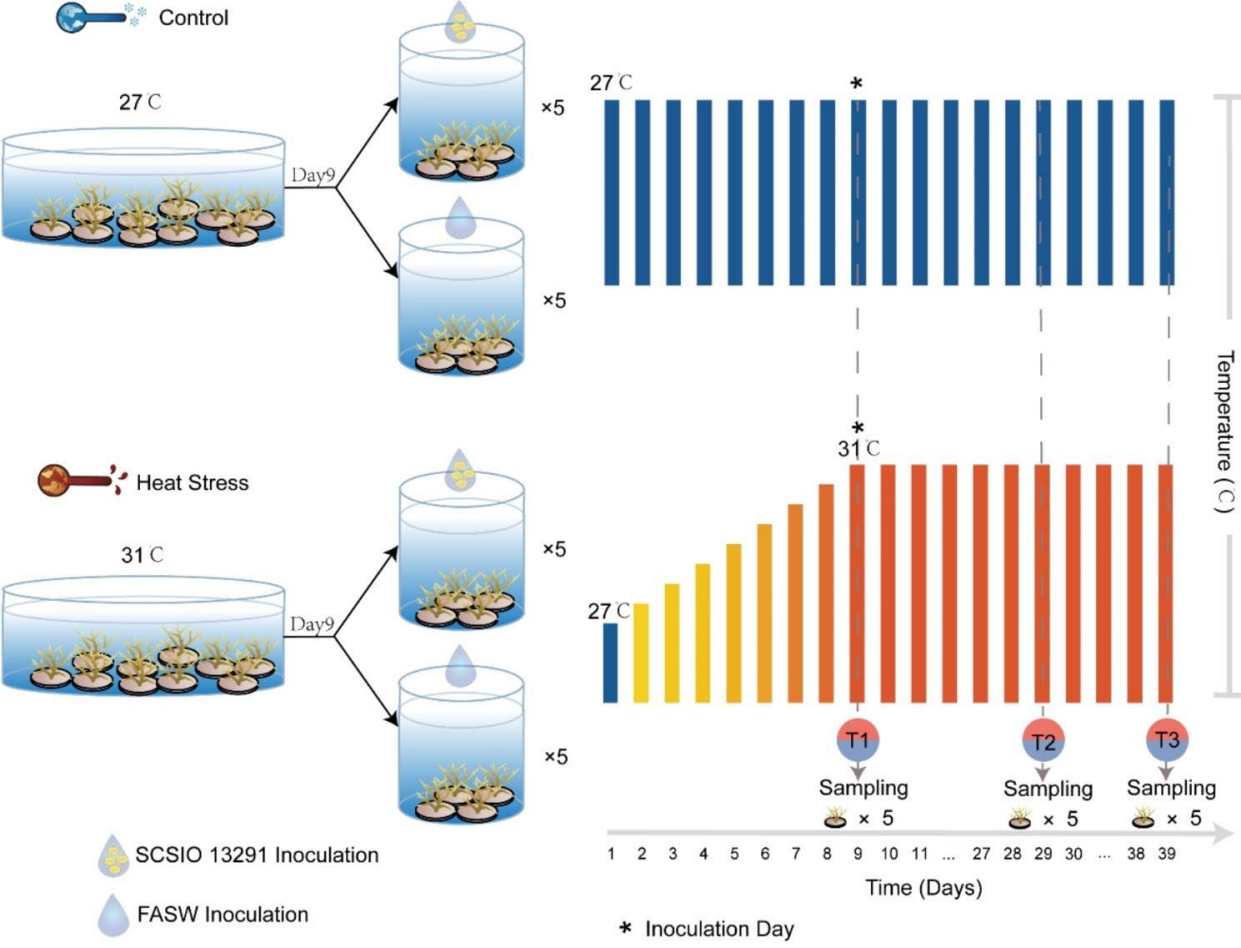
The experimental design, temperature conditions, inoculation and sampling scheme

**Fig. 2:**
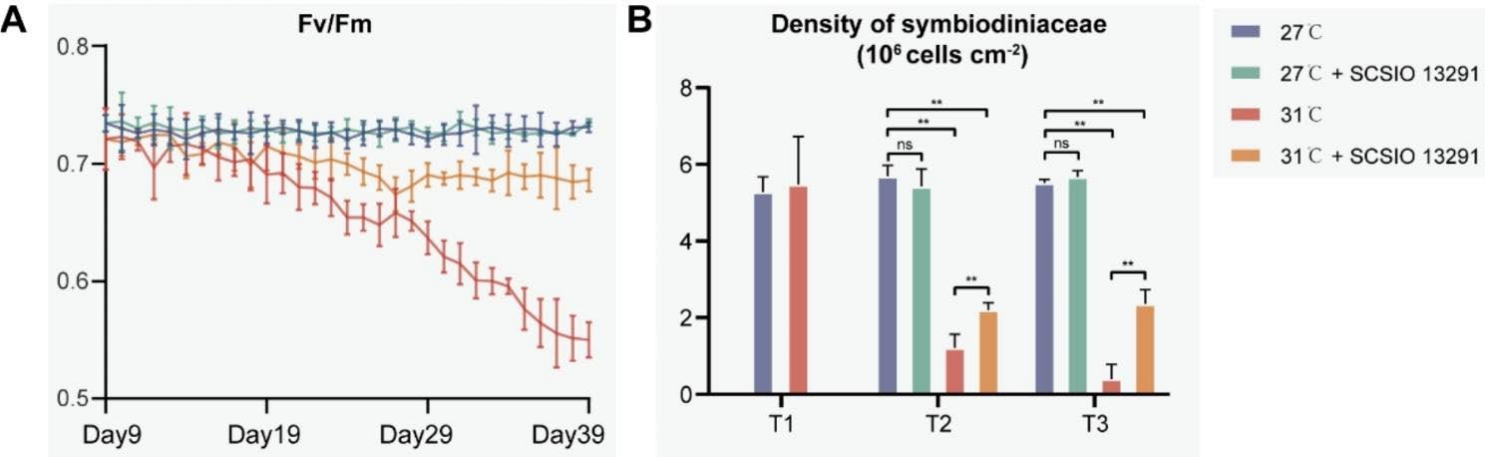
Health status of coral holobionts. (**A**) Measurements of photosynthetic efficiency *Fv/Fm* ratios in *Pocillopora damicornis* at 27 and 31 °C after treatment with strain SCSIO 13291 or FASW until the end of the experiment (n = 10). (**B**) Density of Symbiodiniaceae cells in coral tissue. Numbers are shown as the means ± standard deviations, which were calculated based on five coral nubbins. Asterisks indicate significant differences at *P* < 0.01

The visual appearance of coral nubbins, Symbiodiniaceae density, and photosynthetic efficiency of *P. damicornis* fragments inoculated with strain SCSIO 13291 were compared to control fragments inoculated with 0.2-µm-filtered autoclaved artificial seawater (FASW) (Fig. 1 and Figure S2). The SCSIO 13291-treated (LTB) and FASW-treated (LT) coral nubbins maintained at 27 °C showed *Fv*/*Fm* values between 0.72 and 0.74 and retained Symbiodinaceae densities between 5.29 and 5.69 × 10^6^ cells cm^− 2^ during the 39-day experimental period (Fig. 2A, B). The coral nubbins subjected to thermal stress (31 °C, HT) displayed visual signs of tissue paling with a 25% decrease in the *Fv*/*Fm* average photosynthetic efficiencies rates (*P* < 0.01) at the end of the experiment (T3-day 39) compared to the corals maintained at 27 °C (LT and LTB, Fig. 2A) and Symbiodinaceae densities in coral tissue were 0.40 ± 0.34 × 10^6^ cells cm^− 2^ (Fig. 2B). In contrast, *Fv*/*Fm* average rates of the coral nubbins subjected to thermal stress and inoculated with SCSIO 13291 (HTB) only decreased by approximately 6% (T3) (Fig. 2A). However, the density of Symbiodiniaceae cells in coral tissues did decrease by 57% in the SCSIO 13291-treated corals at 31 °C (HTB, 2.37 ± 0.33 × 10^6^ cells cm^− 2^) though this was still significantly higher (*P* < 0.01) than the corals without inoculation of the bacterial strain (HT, 0.40 ± 0.34 × 10^6^ cells cm^− 2^).

### Changes in bacterial community structure associated with the strain SCSIO 13291 treatment during heat stress

A total of 818,856 high quality 16S rRNA gene sequences were recovered from *P. damicornis* tissue samples derived from 50 libraries which included nubbins maintained at 27 °C (n = 5 replicates for each treatment of T1-day 9 [LT], plus T2-day 29 and T3-day 39 [FASW- and SCSIO 13291-treated samples, respectively referred to LT and LTB groups]) and 31 °C exposed nubbins (n = 5 replicates for each treatment of T1-day 9 [HT], plus T2-day 29 and T3-day 39 FASW- and SCSIO 13291-treatments [referred to HT and HTB group, respectively]). The Amplicon Sequence Variant (ASV) feature table was subsampled with results showing that high temperature significantly shifted the community structure of host-associated bacteria (Figure S3) (T1, permutational multivariate analysis of variance [PERMANOVA]: *F* = 1.963, R^2^ = 0.197, *P* = 0.028; T2, PERMANOVA: *F* = 1.258, R^2^ = 0.136, *P* = 0.028; T3, PERMANOVA: *F* = 1.325, R^2^ = 0.142, *P* = 0.009). The bacterial community structures of samples with FASW-treatment (LT) and SCSIO 13291-treatment (LTB) maintained at 27 °C were similar (T2, PERMANOVA: *F* = 0.966, R^2^ = 0.108, *P* = 0.501; T3, PERMANOVA: *F* = 1.029, R^2^ = 0.114, *P* = 0.336). In addition, the bacterial community structures of SCSIO 13291-treated corals (HTB) were significantly different from FASW-treated corals (HT) at 31 °C (T2, PERMANOVA: *F* = 1.848, R^2^ = 0.188, *P* = 0.028; T3, PERMANOVA: *F* = 1.996, R^2^ = 0.200, *P* = 0.009) and SCSIO 13291-treated corals (LTB) at 27 °C (T2, PERMANOVA: *F* = 2.525, R^2^ = 0.240, *P* = 0.021; T3, PERMANOVA: *F* = 2.181, R^2^ = 0.214, *P* = 0.016) (Figure S3).

**Fig. 3:**
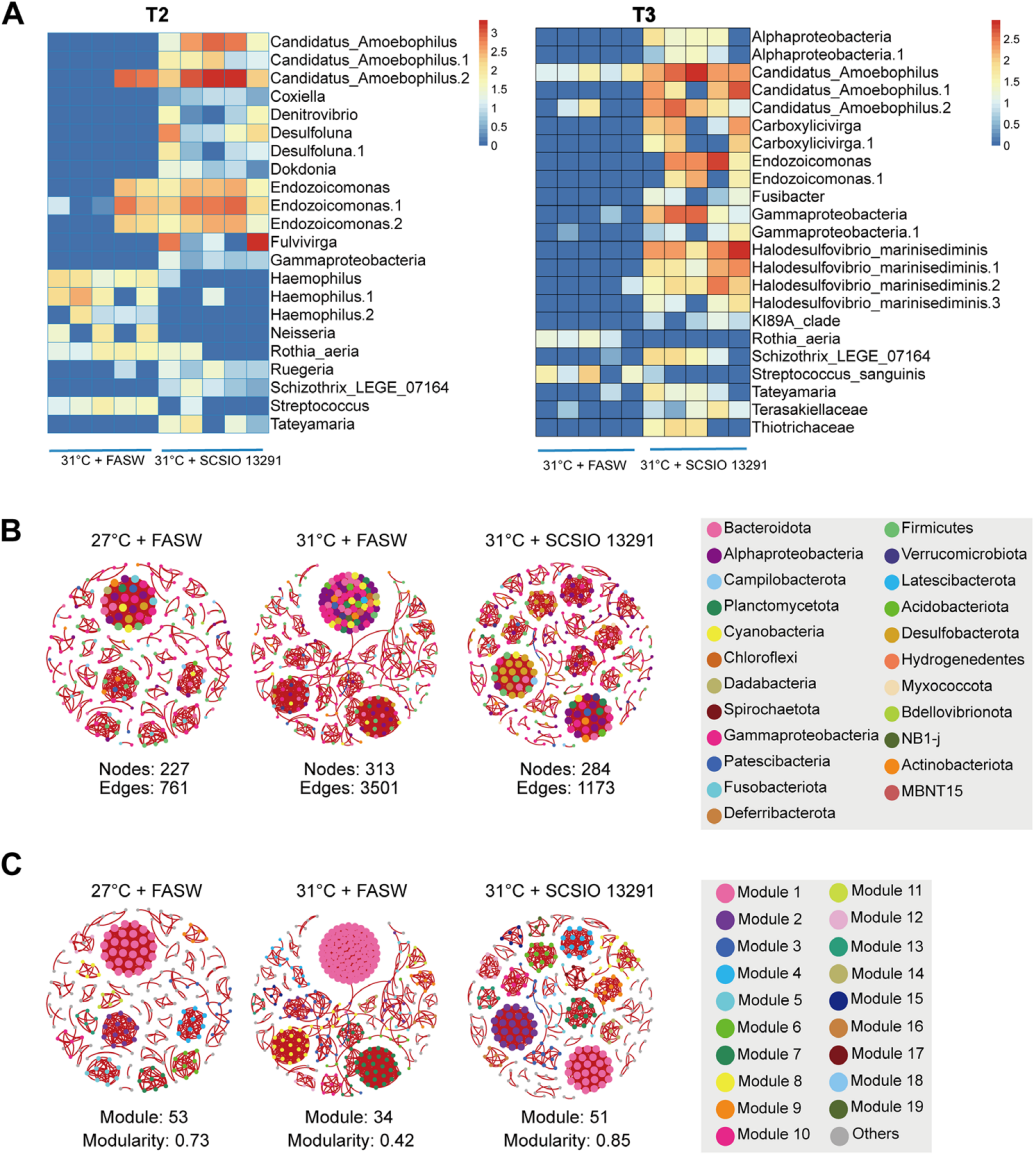
Differential ASVs and networks of *Pocillopora damicornis* -associated bacterial communities. (**A**) ASVs with differential relative abundances (FDR < 0.05) between samples inoculated with strain SCSIO 13291 and FASW at 31 °C. Taxonomic annotations of each ASV are also shown in the figure. (**B**) An overview of bacterial networks colored by corresponding phylum/class. (**C**) An overview of bacterial networks colored by corresponding modules. Red edges indicate positive relationships, and blue edges indicate negative relationships. Modules with more than 5 nodes are indicated using different colors

Many ASVs exhibited differences in relative abundance between treatments (Fig. 3A). For example, ASVs belonging to candidatus *Amoebophilus*, *Endozoicomonas*, *Fulvivirga*, *Desulfoluna*, *Halodesulfovibrio marinisediminis*, and *Carboxylicivirga* were significantly enriched in the SCSIO 13291-treated coral nubbins (HTB) under heat stress while the relative abundances of the ASVs identified as members of *Haemophilus*, *Rothia*, *Streptococcus*, and *Neisseria*, which have been previously been linked with human [26], reef fish [27, 28], and coral [29, 30] diseases were significantly decreased. Propionibacteriaceae affiliated sequences were retrieved from 75% of the samples across all treatments, though the relative abundance of these sequences was generally less than 1% and did not show significant differences between treatments. The retrieved Propionibacteriaceae affiliated sequences also only displayed between 90 and 94.1% similarity to the 16S rRNA gene sequence derived from strain SCSIO 13291. Despite not retrieving sequences unambiguously affiliated to the strain SCSIO 13291, fluorescence signals of 5 (and − 6)-carboxytetramethylrhodamine, succinimidyl ester (TAMRA/SE) were observed near the oral disk and attached to the column of the coral polyp at T2 and T3 timepoints under 31 °C (Figure S4), but it was unclear whether the labeled bacteria were on the surface or beneath the body wall. No TAMRA/SE fluorescence detection signal was observed in the coral polyps visualized before inoculation (T1) or in control treatments with added FASW (T2 and T3).

Bacterial co-occurrence networks explored the patterns in interactions in the corals maintained at normal temperature (27 °C, LT, labeled as 27 °C + FASW; Fig. 3B and C), under heat stress (31 °C, HT, labeled as 31 °C + FASW; Fig. 3B and C), and inoculated with SCSIO 13291 (31 °C, HTB, labeled as 31 °C + SCSIO 13291; Fig. 3B and C). The main taxa in the LT, HT and HTB networks were different, especially in the HTB network, where more nodes were affiliated with *Alphaproteobacteria* and *Desulfobacterota* (Figure S5). The clustering coefficient and modularity in the empirical networks were higher than those in the respective Erdös–Réyni random networks, which suggested that the networks had small-world properties and modular structures (Table S1). The LT, HT and HTB networks displayed strong differences in their structure and topology (Figure S6, Table S1). The HT network had more nodes and edges than the other networks (Fig. 3B). Moreover, the Kolmogorov–Smirnov test showed that the degree and betweenness centrality were significantly higher in the HT network than in the LT and HTB networks (Figure S6). The LT and HTB networks had a lower average path length and higher small-world coefficient and modularity value than the HT network. According to their within-module connectivity (*Zi*) and among-module connectivity (*Pi*) values, the ASVs were mainly classified into connectors and peripherals (Figure S7). There were 36 connectors in both the LT and HTB networks, and the HT network had 26 connectors; these connectors were mainly from the phyla *Proteobacteria*, *Bacteroidota*, *Firmicutes*, and *Desulfobacterota* (Table S2).

### Transcriptional reprogramming of strain SCSIO 13291-treated coral holobionts

A total of 32,525 coral genes and 43,088 Symbiodiniaceae unigenes were identified from RNA sequencing of coral nubbins maintained at 27 °C (LT group, T1-day 9, plus T2-day 29 and T3-day 39 [FASW-treated samples]) and 31 °C (T1-day 9 [HT], plus T2-day 29 and T3-day 39 [FASW-treated and SCSIO 13291-treated coral nubbins, referred to HT and HTB group, respectively]). Significant differences in coral expression profiles between the 27 °C (LT) and 31 °C treatments (FASW-treated, HT) were observed at the T2 and T3 timepoints (*P* < 0.05, PERMANOVA; Figure S8), though not at the T1 timepoint (*P* = 0.07, PERMANOVA; Figure S8). At timepoints T2 and T3, the coral genes were significantly differentially expressed between the strain SCSIO 13291-treated (HTB) and FASW-treated (HT) coral nubbins maintained at 31 °C (*P* = 0.0195, T2; *P* = 0.033, T3) and between the SCSIO 13291-treated corals (HTB) cultured at 31 °C and the controls (LT) cultured at 27 °C (*P* = 0.044, T2; *P* = 0.033, T3; Figure S8). At both timepoints T2 and T3, the Symbiodiniaceae genes were significantly differentially expressed between the SCSIO 13291-treated (HTB) and FASW-treated (HT) coral nubbins cultured at 31 °C (*P* = 0.024, T2; *P* = 0.045, T3; Figure S9).

We further investigated the differentially expressed genes of the SCSIO 13291- and FASW-treated nubbin samples maintained at 31 °C. Strain SCSIO 13291-treated corals (HTB) had 2818 and 3311 up- and down-regulated genes, respectively, with a false discovery rate (FDR) of < 0.05 at T2 timepoint (day 29) compared to FASW-treated corals (HT); and 2466 and 2358 up- and down-regulated genes (FDR < 0.05), respectively, at T3 (day 39). The downregulated genes were significantly enriched in gene ontology (GO) categories mainly related to signal transduction, apoptosis regulation, and transcription regulation. Moreover, many downregulated genes observed at T2 were related to exocytosis (Figure S10). The upregulated genes were significantly enriched in GO categories mainly related to ribosome, translation, cell redox homeostasis, ATP synthesis, proteasome, protein folding, chaperone binding, methylation, and DNA repair (Figure S10).

For the endosymbiotic algae, the samples (HTB) inoculated with strain SCSIO 13291 had 2081 and 2940 up- and down-regulated genes (FDR < 0.05), respectively, at 31°C (T2-day 29) compared to FASW-treated corals (HT); and 1124 and 1002 up- and down-regulated genes (FDR < 0.05), respectively, at T3 timepoint (day 39). The downregulated genes were significantly enriched in GO categories mainly related to carbon fixation, ribulose-bisphosphate carboxylase activity, nitrate assimilation, signal transduction, and ion transport (Figure S11). The upregulated genes were significantly enriched in GO categories mainly related to photosynthesis, photosystem I and II, thylakoid membrane, light-harvesting complex, ATP synthesis, proton transmembrane transporter activity, and respirasome (Figure S11).

**Fig. 4:**
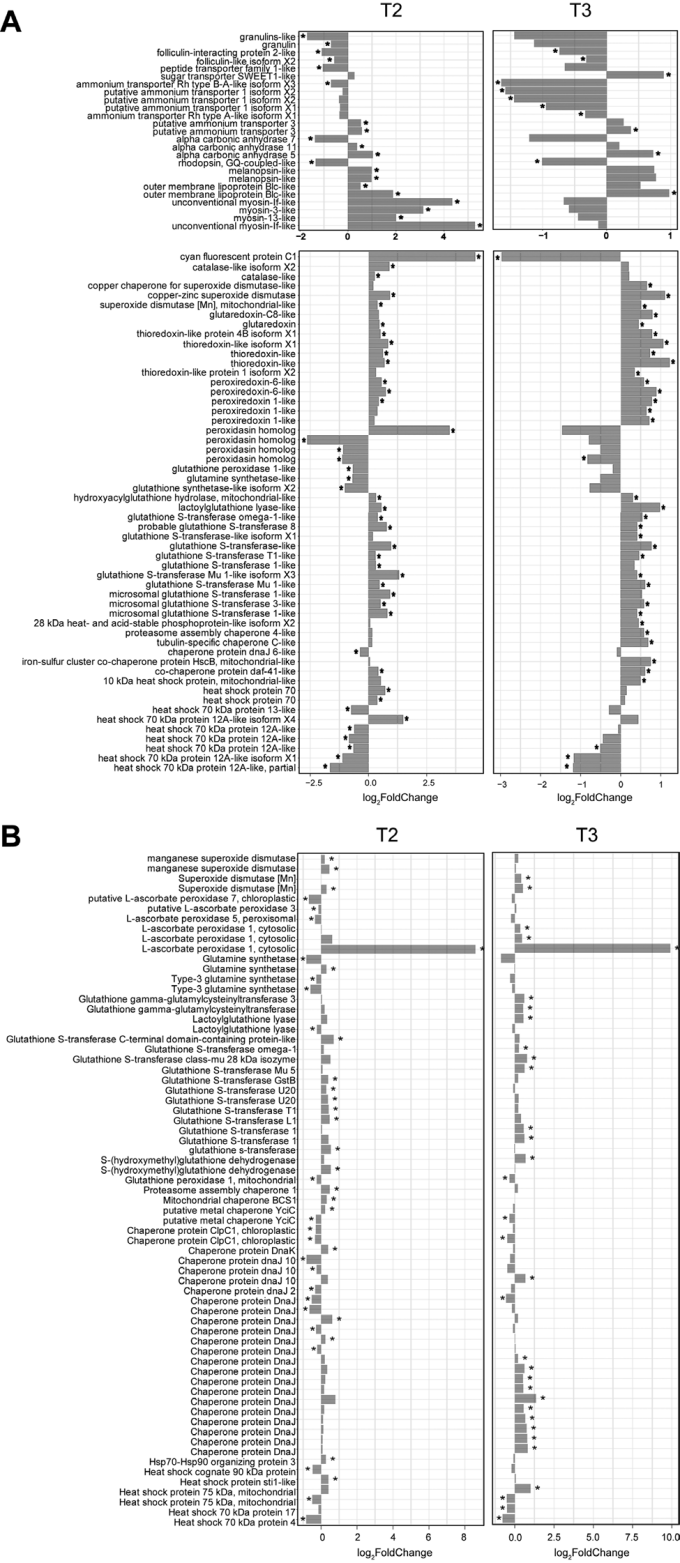
Differentially expressed genes between coral holobionts inoculated with strain SCSIO 13291 and FASW at 31 °C. (**A**) Expression profiles of the specific genes involved in symbiosis, heat stress response, and bleaching tolerance in coral hosts. (**B**) Expression profiles of the specific genes known to be involved in the heat stress response and tolerance in Symbiodiniaceae. Asterisks indicate significant differences at FDR < 0.05. Log_2_FoldChange represents upregulation or downregulation in relation to SCSIO 13291 treatment

We also explored the expression profiles of the genes known to be involved in symbiosis, heat stress response, and bleaching tolerance [6, 31] through comparing SCSIO 13291-treated (HTB) and FASW-treated (HT) corals at 31 °C. Genes encoding putative myosin-like protein were upregulated (log_2_FoldChanges [log_2_FCs] from 2.0 to 5.3, FDR < 0.05) at T2 but downregulated though not significantly (log_2_FCs from − 0.1 to -0.7, FDR > 0.05) at T3 in coral hosts (Fig. 4A). The lipid transporter, Blc-like outer membrane lipoprotein, was overexpressed in coral hosts inoculated with SCSIO 13291 (Fig. 4A). The alpha carbonic anhydrase 5 was overexpressed in SCSIO 13291-treated coral hosts, while alpha carbonic anhydrase 7 was suppressed (Fig. 4A). Overall, more ammonium transporters were downregulated in coral hosts treated with SCSIO 13291 at the T3 timepoint. The sugar transporter SWEET1-like was upregulated in coral hosts, while the peptide transporter family 1-like and the folliculin complex (folliculin and folliculin-interacting proteins [FNIP]) involved in sensing amino acid availability [31] were downregulated. Two melanopsin-like genes were upregulated in SCSIO 13291-treated coral hosts (log_2_FCs from 0.7 to 1.0), although not significantly at T3, and one rhodopsin, CQ-coupled-like, was downregulated (log_2_FCs from − 1.0 to -1.3, FDR < 0.05). Granulins, which were found to be expressed in alga-containing gastrodermal cells [32], were downregulated in SCSIO 13291-treated corals (Fig. 4A). An additional 56 differentially expressed tetratricopeptide repeat protein 28-like genes, were mostly downregulated in SCSIO 13291-treated coral hosts (only four of them were significantly upregulated at T2) (Figure S12). Most of the heat stress response and bleaching tolerance related genes were upregulated in SCSIO 13291-treated coral hosts collected at T2 and T3 timepoints, such as glutathione S-transferases, peroxiredoxin, thioredoxin, glutaredoxin, superoxide dismutases, and catalase, while glutamine synthetase-like and glutathione synthetase-like isoform X2 were less expressed (Fig. 4A). Additionally, cyan fluorescent protein (CFP) was overexpressed (log_2_FCs = 4.6, FDR = 0.01) in the SCSIO 13291-treated coral hosts at T2 but downregulated (log_2_FCs = -3.0, FDR = 0.01) at T3. Glutathione S-transferases, glutathione gamma-glutamylcysteinyltransferase, L-ascorbate peroxidase 1 (cytosolic) and manganese superoxide dismutase were overexpressed in endosymbiotic algae in SCSIO 13291-treated samples (Fig. 4B). In particular, one L-ascorbate peroxidase 1 (cytosolic) was highly upregulated in endosymbiotic algae in SCSIO 13291-treated nubbins, with log_2_FCs = 8.6 and 9.9 at T2 and T3 (FDR < 0.01), respectively (Fig. 4B).

## Discussion

Inoculation of the actinobacterial strain SCSIO 13291 to thermally stressed coral nubbins partially mitigated the visual signs of bleaching and maintained higher Symbiodiniaceae cell densities and photosynthetic *Fv*/*Fm* average rates relative to untreated corals. These effects were similar to previous reported studies that inoculated bacterial consortia onto heat stressed or pathogen challenged coral, thereby conferring benefits to the coral host [18, 19]. The mechanism by which the inoculated bacteria increase the resilience of the coral host to stress or challenge is unknown though the associated bacterial communities restructuring and coral transcriptional reprogramming were observed [18, 19].

Inoculated bacteria can potentially directly benefit the coral through conferring metabolic traits that boost holobiont performance or through indirect mechanisms of suppling additional nutrient sources that promote symbiont growth and associated coral health indicators. The SCSIO 13291 strain inoculated in this study was labeled with the vital fluorescent stain TAMRA/SE, which has no adverse effects on cell viability and has been used in monitoring bacterial transport in subsurface environments [33] and hemocytic phagocytosis of bacteria [34]. Microscopy images of coral polyps detected fluorescent signals consistent with TAMRA/SE stained bacteria near the oral disk of the polyp and attached to the column of inoculated coral samples collected at both T2 and T3 timepoints, indicating that the inoculated bacterial strain is associated with the corals throughout the experimental period. However, the Propionibacteriaceae affiliated 16S rRNA gene sequences recovered from the inoculated corals represented less than 1% of the total sequences retrieved and representative ASVs displayed only 90–94.1% similarity to the 16S rRNA gene sequence of the inoculated SCSIO 13291 strain. Previous studies that have inoculated bacteria to coral (BMC cocktails) similarly reported low recovery of 16S rRNA gene sequences affiliated to the inoculated strains [18, 19, 35]. Hence results based on 16S rRNA gene sequencing suggest that the inoculated strains may not establish an association with the corals nubbins, though amplicon sequencing also has limitations including biases associated with DNA extraction and amplification [36]. Elucidating if the inoculated bacteria establish an association with the coral host, localizing where these association are, how long they are maintained and subsequently characterizing if metabolic activity of the added bacteria directly benefit the coral host is essential in future studies [21]. Alternatively, the inoculation of bacteria may provide a short-term stimulatory benefit to the coral holobiont through nutrient acquisition, modulating the associated bacterial community structure plus Symbiodiniaceae and coral host metabolic activity.

Inoculation of the SCSIO 13291 strain resulted in significant changes in the coral associated microbial structure, consistent with previous studies profiling the bacterial communities of BMC-treated corals [18–20]. Network analyses identified that the modularity of the interaction between bacterial communities associated with FASW-treated corals at 31 °C was lower (less stable [37, 38]) than those associated with corals maintained at 27 °C and SCSIO 13291-treated corals at 31 °C, although the average degree and link were higher. These alterations might be induced by additional heterotrophic nutrients supplied by dosing the SCSIO 13291 strain to the corals or alternatively through specific active metabolites produced by the SCSIO 13291 strain. Although cooccurring networks of the coral microbiome have been elucidated across different treatment scenarios [39–41], the relationship between the tissue specific bacterial interactions and the resilience and tolerance of coral holobionts subjected thermal stress is complicated, though crucial for understanding the resilience of coral holobionts to ocean warming.

At the ASVs level, several potential pathogens were significantly decreased in relative abundance in the SCSIO 13291 inoculated coral nubbins under thermal stress, while ASVs affiliated with *Endozoicomonas* and Candidatus *Amoebophilus* (T2 & T3) were enriched. *Endozoicomonas* affiliated taxa often dominant coral microbiomes, accumulated in aggregates (Cell Associated Microbial Aggregates) within the coral tissues and proposed to be a beneficial symbiont [42–46]. However, under environmental stress the relative abundance of *Endozoicomonas* taxa decrease likely through dysbiosis processes [7, 47, 48]. The maintenance of *Endozoicomonas* population in the tissues of the SCSIO 13291 inoculated corals likely benefited the coral holobiont during the heat stress treatment. The Bacteroidetes genus “Candidatus *Amoebophilus*” is of interest due to characteristic endosymbiotic traits, including smaller genomes and associated reduced metabolic capabilities while also harboring a high count of host cell interaction genes [49–52]. Its role in the coral holobiont, especially enriched in the SCSIO 13291 inoculated corals which displayed better physiological status under temperature stress, is unclear. Additionally, several ASVs enriched in SCSIO 13291-treated samples under thermal stress, including an unknown Gammaproteobacteria, *Denitrovibrio*, *Fulvivirga*, *Coxiella*, and *Halodesulfovibrio marinisediminis*, were also determined to be keystone species in the co-occurrence networks. Enrichment of *Denitrovibrio* might enhance nitrate reduction to ammonium [53], and thus supply the preferred nitrogen source to Symbiodiniaceae [54], which may be linked to the downregulation of ammonium transporters in the coral host. *Fulvivirga* affiliated taxa, like *Endozoicomonas* have been found to be more abundant in healthy corals [55], while *Coxiella* has previously been reported as a common coral associated symbiont [56]. A novel member of the sulfate-reducing bacterium *Halodesulfovibrio* has been proposed to syntrophically interact with coral-associated *Prosthecochloris*, which is capable of carbon and nitrogen fixation in coral skeletons [57]. However, little information is known about the potential function of this genus in coral tissues in addition to many of the other affiliated taxa recovered in this study. Given their potential important roles identified within the network links of the coral microbiome, further investigations are required to establish their roles in structuring the coral-associated bacterial community and influence on thermal tolerance.

Transcriptional profiling demonstrated significant differences in gene expression patterns between SCSIO 13291-treated corals maintained at 31 °C and FASW-treated corals subjected to 27 and 31 °C. Photosystem and photosynthesis-associated genes were upregulated in Symbiodiniaceae of coral nubbins inoculated with SCSIO 13291, especially at T3, and the *Fv*/*Fm* rates were higher than those of the FASW-treated corals (albeit lower than corals at 27 °C) suggesting mitigation of damage to the photosystems for Symbiodiniaceae in inoculated corals [19, 58]. Several genes putatively involved in apoptosis and exocytosis were also downregulated in the SCSIO 13291 inoculated coral hosts, correlating with these higher Symbiodiniaceae densities. Apoptosis and exocytosis are the processes through which corals expel algal symbionts under thermal stress and ultimately manifest a bleaching phenotype [3, 4]. Therefore, inoculation of the bacterial strain potentially resulted in less damaged Symbiodiniaceae photosystems and downregulated putative apoptosis- and exocytosis-related proteins of the coral host which together mitigated Symbiodiniaceae loss. The underlying mechanisms for these responses are unknown however, and maybe the result of release of the Symbiodiniaceae from nutrient limitation within the coral gastrodermal cell layer [5, 59]. This is somewhat supported through analysis of the metabolite transportation expression patterns. We found the downregulation of ammonium and peptide transporters and the folliculin complex involved in sensing amino acid availability and the upregulation of the sugar transporter SWEET1-like in SCSIO 13291-treated coral hosts. The peptide transporter and folliculin complex were previously found to be overexpressed in alga-hosting cells in single–cell transcriptional analysis [31]. The SCSIO 13291-treated coral may transfer available sugars supplied by the dosed bacteria rather than peptides or amino acids derived from algal symbionts.

Expression of peroxiredoxin, thioredoxin, glutaredoxin, superoxide dismutases and catalase associated genes were upregulated in SCSIO 13291-treated coral hosts, while ascorbate peroxidase and manganese superoxide dismutase were upregulated in Symbiodiniaceae. Hence actinobacterial inoculation appeared to enhance activity of these antioxidants, which could protect corals and Symbiodiniaceae from tissue damage caused by reactive oxygen species (ROS) under thermal stress. Strain SCSIO 13291 possessed catalase activity and the capability to synthesize heme, both of which potentially functioned in ROS elimination. In addition, strain SCSIO 13291 has the ability synthesize nisin and labyrinthopeptin compounds with demonstrated antibacterial and antivirus activities [60] and which may contribute to the defense against pathogens and viruses in the coral holobiont. The presence of these antioxidative and antimicrobial capabilities supports the beneficial effects of SCSIO 13291, though further investigations are required to determine if these bioactive compounds are their associated specific activities are conferred directly to the coral holobiont and maintained over time to mitigate stress.

**Fig. 5:**
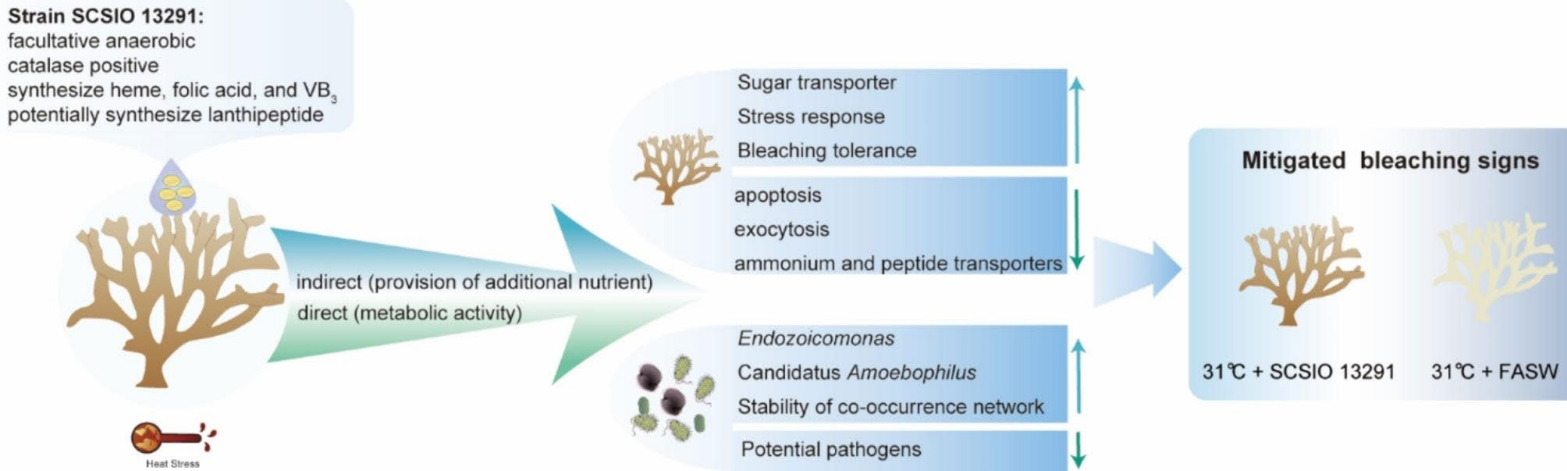
Summary of the alterations in bacterial community and expression profile in strain SCSIO 13291-treated coral samples that showed mitigated bleaching signs. We proposed that these responses could be results of nutrients supplied through inoculation of the bacterium (indirect mechanism) or metabolic activity of the added bacterium associating with the coral polyp (direct mechanism)

In the face of global climate change, the tolerance of coral holobionts to heat stress is one key factor to their long-term survival [61]. The results of this study showed that inoculation of an actinobacterial strain can mitigate coral bleaching signs and this was accompanied by alterations in the coral-associated bacterial community and their ecological networks. A suite of coral host and Symbiodiniaceae responses were observed in SCSIO 13291-treated samples, including protection from ROS and light damage, attenuated apoptosis and exocytosis, and metabolites utilization, potentially underlying coral thermal tolerance. These responses across the coral host, Symbiodiniaceae, and associated bacterial community could be result of nutrients supplied through inoculation of the bacteria or through direct metabolic activity of the bacteria associating with the coral polyp (Fig. 5). It is worthy to include the killed bacterium in the future experiment to acquire more evidence [62]. Irrespective of these direct or indirect mechanisms, our results provide insights into the beneficial effects of a putative actinobacteria coral probiotic and supports the feasibility of enhancing coral resistance to adverse environmental conditions through modulation of the associated microbiome.

## Materials and methods

### Isolation, identification, and characterization of strain SCSIO 13291

Strain SCSIO 13291 was isolated from tissues of the healthy coral *P. damicornis* collected from the Luhuitou fringing reef (109°29′ E, 18°13′ N) at Sanya, China, in November 2012. Briefly, the coral fragment was rinsed with sterile artificial seawater (sea salts; Sigma‒Aldrich, USA) to remove loosely attached bacteria, and then tissues were removed from the coral skeleton using sterile artificial seawater that was sprayed using a syringe. The obtained tissue slurry was serially diluted and spread on plates containing glutamine agar medium (0.5 g glutamine, 15 g agar, and 1 L 0.2-µm-filtered natural seawater). The plates were incubated at 25 °C under aerobic conditions for 30 days. Single colonies were picked and streaked onto new plates to obtain pure cultures. The genome sequencing and processing, phylogenetic analysis, physiological and biochemical tests, and vitamin assays were performed according to the methods described in our previous report [63] (supplementary Dataset S2). Heme assay was performed using a fluorometric method [64]. The strain was stored in a glycerol suspension (20%, v/v) at − 80 °C and as freeze-dried cultures at 4 °C.

### Strain SCSIO 13291 growth and labeling

Strain SCSIO 13291 was recovered from a -80 °C glycerol stock on trypticase soy agar (TSA; Becton, Dickinson and Co.) prepared with artificial seawater (sea salts; Sigma‒Aldrich, USA), and a single colony was transferred to 5 ml of trypticase soy broth (TSB; Becton, Dickinson and Co., BD) prepared with artificial seawater and shaken at 28 °C and 180 rpm for 48 h. Subsequently a 4 ml aliquot of the culture was added to 200 ml TSB medium prepared with artificial seawater and incubated at 28 °C and 180 rpm for 48 h. The SCSIO 13291 cells were collected after centrifugation at 4500 *g* for 15 min and washed twice with 0.2-µm-filtered autoclaved artificial seawater (FASW), then resuspended in FASW at a cell density of approximately 10^7^ cells ml^− 1^. The concentrated cell suspension was stained with 5 (and − 6)-carboxytetramethylrhodamine, succinimidyl ester (TAMRA/SE), for 30 min at a final concentration of approximately 150 µmol/L without light. The effect of TAMRA/SE on the physiological and biochemical properties was determined using the BIOLOG GEN III MicroPlate (Biolog Inc.), and the labeled and unlabeled cells showed identical results (Table S3).

### Sample collection

One *P. damicornis* colony was collected from the Luhuitou fringing reef (109°28′ E, 18°13′ N) at Sanya, China, in August 2020 and placed in a 30 L plastic box containing natural seawater. At the time of sample collection, the seawater temperature was 28.4 °C and the salinity was 32‰. Upon arrival at the indoor laboratory in Guangzhou approximately 20 h after collection, the coral colony was transferred to a 200 L aquarium with circulating artificial seawater prepared by mixing deionized water with Reef Crystals sea salt (Aquarium Systems). The water temperature was 26–27 °C, the salinity was 31–33‰, and the light intensity was approximately 150 µmol photons m^− 2^ s^− 1^ in a 12:12-h light/dark cycle. After five days of acclimation, the colony was divided into ~ 90 nubbins (ca. 2–3 cm high), which were then attached to ceramic reef discs using glue (aron alpha GEL-10) and the nubbins allowed to acclimate for a further three days.

### Heat stress experiment

The coral nubbins were randomly assigned to two 60-L tanks, with 45 nubbins per tank (Fig. 1; day 1). The water temperature was maintained at 27–27.5 °C in the control tank and was increased by 0.5 °C per day to 31 °C in the experimental tank. After reaching the peak temperature (31 °C) on day 9, five coral nubbins were randomly collected from the control and experimental tank, respectively, and then half of the nubbins in each tank were inoculated with strain SCSIO 13291 labeled with TAMRA/SE, and the other nubbins were inoculated with FASW as placebo. Inoculation was conducted according to the following procedure: (1) one nubbin was placed in a 250 mL sterile plastic culture bottle, then 1 mL of fluorescently labeled bacterial cell suspensions (approximately 10^7^ cells) or FASW was slowly dropped onto the coral surface exposed to air; (2) one hundred milliliter of sterilized seawater collected from the culture system was added to the culture bottle making the fragments completely submerged, then the bottle was put back into the original tank; (3) after keeping in the culture bottle for 45 min, the inoculated nubbin was taken out and put into a new experimental tank, in which the temperature, pH, and salinity of the water were the same as the original tank. The coral nubbins inoculated with strain SCSIO 13291 or FASW were divided into five tanks at each temperature. The experiment was carried out for a total of 39 days. The water temperature of each tank was regulated using a submersible aquarium heater (EHEIM, GmbH & Co. KG, Deizisau, Germany).

Five coral nubbins were randomly collected at day 9 (before inoculation, T1), day 29 (T2), and day 39 (T3). Selected coral nubbins were removed from the ceramic reef discs and gently washed using FASW. Each nubbin was then cut into three fragments using a sterilized bone cutter. One fragment of each nubbin was placed in a Falcon tube on ice and used to determine the abundance of Symbiodiniaceae cells within 30 min after sampling. The remaining fragments were immediately placed into a 50-mL Falcon tube containing enough RNA*later* solution (QIAGEN GmbH, Hilden, Germany) to completely submerge the fragments. After being refrigerated overnight at 4 °C, the samples were stored at -30 °C until DNA or RNA extraction.

### Assessment of coral health

During the experiment, each nubbin was photographed daily with a Olympus TG-4 camera under the same conditions. The photosynthetic efficiency of Symbiodiniaceae was assessed daily using a submersible diving-pulse amplitude-modulated (PAM) system (Walz GmbH, Effeltrich, Germany). Measurements were performed after at least 30 min in the dark to avoid non-photochemical dissipation of excitation energy by photosystem II (PSII). The maximum quantum yield of PSII photochemistry was determined as *F*_*v*_*/F*_*m*_. The diving-PAM was configured according to previous studies [18, 19]. Ten nubbins from each experimental group (i.e., 27 °C, LT; 27 °C + SCSIO 13291, LTB; 31 °C, HT; 31 °C + SCSIO 13291, HTB) were tested randomly, and three positions on each nubbin were tested.

The cell abundance of Symbiodiniaceae was assessed according to the method described previously [7] with a minor modification that five replicate counts using five droplets of 10 µL each were performed per sample. The coral surface area was measured using the aluminum foil coating method [65], and the density of Symbiodiniaceae was calculated as the average number of cells per cm^2^ coral surface area.

### Host-SCSIO 13291 visualization

Small branch tips were clipped from the coral nubbins collected at the three timepoints (T1, T2, and T3). After gently washing the surface with FASW, the fragments were placed in sterile 4-well chambered coverglass (Nunc™ Lab-Tek™, ThermoFisher Scientific) containing 500 µL of FASW. The autofluorescence of coral tissue (excitation: 488 nm, emission: 525 nm) and Symbiodiniaceae (excitation: 488 nm, emission: 679 nm) and the TAMRA/SE signal (excitation: 551 nm, emission: 578 nm) were observed using a confocal laser scanning microscope (Leica TCS SP8, Leica, Wetzlar, Germany).

### DNA and RNA extraction

Tubes containing coral fragments were put on ice, samples were picked up from RNA*later* solution using sterilized forceps, and excess solution was removed using kimwipes [7]. Genomic DNA was extracted using a PowerSoil® DNA Isolation Kit (MO BIO, Carlsbad, CA, USA). Three replicates of blank extractions (i.e., without coral samples) were produced simultaneously. After votexing the powerbead tubes (coral fragments were added) using a Vortex-Genie 2 (MO BIO) at the maximum speed for 10 min, tissues were removed from the skeleton. After removing the skeleton, the following manipulation was performed according to the protocol provided by the kit. Total RNA was extracted using a RNeasy Mini Kit (QIAGEN GmbH, Hilden, Germany). Initially, the coral fragments were placed in tubes containing glass beads of 2 mm diameter (Sangon Biotech, Shanghai, China) and buffer RLT provided by the RNeasy Mini kit. The tubes were placed on a Vortex-Genie 2 and vortexed at maximum speed for 5 min, and the tissues were consequently removed from the skeleton. After removing the skeleton, the extraction was performed according to the manufacturer’s instructions.

### 16S rRNA gene amplification, sequencing, and analysis

To obtain full-length 16S rRNA gene amplicons, we used the universal primers 27F (5′-AGRGTTTGATYNTGGCTCAG-3′) and 1492R (5′-TASGGHTACCTTGTTASGACTT-3′) tagged with 16-nt PacBio barcodes at the 5′ ends. PCR amplification was performed in triplicate per sample using the KOD One PCR Master Mix (TOYOBO, Osaka, Japan), with approximately 100 ng DNA template and a final primer concentration of 0.2 µmol L^− 1^. The PCR conditions were 95 °C for 2 min, followed by 25 cycles of 95 °C for 10 s, 55 °C for 30 s and 72 °C for 90 s, with a final extension time of 2 min. Triplicate PCR products were pooled and purified using VAHTSTM DNA Clean Beads (Vazyme, Nanjing, China) and a Monarch DNA Gel Extraction Kit (NEB, Ipswitch, MA). Purified PCR products were quantified using the Qubit dsDNA HS Assay Kit (Invitrogen, Oregon, USA). On the basis of the results, barcoded amplicons were pooled in equimolar concentrations with 40 samples per pool. SMRTbell libraries were prepared according to the SMRTbell Template Prep Kit (Pacific Biosciences, Menlo Park, USA) instructions and sequenced on PacBio Sequel II platform (Pacific Biosciences, Menlo Park, USA).

Circular consensus sequencing (CCS) reads were obtained from PacBio raw data using the SMRT Link software v8.0 with the minPasses ≧ 5 and minPredictedAccuracy ≧ 0.9. The CCS reads were assigned to the corresponding samples using lima (v1.7.0) based on the barcodes. Identifying and removing primers and filtering CCS reads that out of the length range (1,200-1,650 bp) were performed by using Cutadapt v2.7 [66]. The CCS reads were then processed using QIIME 2 Pipeline v2020.02 [67]. The plugin Demux was used to create interactive quality plots and assess the quality. Plugin DADA2 [68] was used for denoising and chimera checking and generation of a feature table. Taxonomy was assigned to amplicon sequence variants (ASVs) using the q2-feature-classifier [69], which employs the classify-sklearn naïve Bayes taxonomy classifier against the SILVA 138 99% operational taxonomic units (OTUs) full-length reference sequences [70]. Non-target ASVs, such as chloroplasts, mitochondria, archaea, eukaryotes, and unidentified ASVs, and ASVs with relative abundance of less than 0.005% [71], were excluded from the final ASV table. Statistical analysis was conducted on the basis of the ASV data. Non-metric multidimensional scaling (nMDS) was performed according to the Bray‒Curtis distance matrix. Permutational multivariate analysis of variance (PERMANOVA) was used to test the differences between microbial communities. NMDS and PERMANOVA analyses were performed using the vegan program packages (version 2.5-6) in R [72]. Differential taxa between samples were determined using the ALDEx2 package (version 1.22.0) in R [73].

## Co-occurrence network analyses

To examine how high temperature and inoculated bacterial strain affect the co-occurrence patterns of bacterial communities, co-occurrence network was constructed by calculating pairwise Spearman rank correlations (Spearman’s rho) on the basis of the bacterial communities in coral samples collected at 27 °C (FASW-treated, LT) and 31 °C (SCSIO 13291-treated, HTB; FASW-treated, HT) at the T2 and T3 timepoints. The ASVs found in at least 30% of samples were used to construct the networks. Valid co-occurrences with |rho| > 0.7 and *P*-value < 0.05 were incorporated into the networks. We assessed the role of network nodes by the within-module connectivity (*Zi*) and among-module connectivity (*Pi*), which were defined according to the connectivity of each node [74]. The node topologies are divided into four categories: network hubs (*Zi* > 2.5 and *Pi* > 0.62), module hubs (*Zi* > 2.5 and *Pi* < 0.62), connectors (*Zi* < 2.5 and *Pi* > 0.62) and peripherals (*Zi* < 2.5 and *Pi* < 0.62). Here, to examine the difference among the three networks, bootstrapping node attributes (degree, betweenness centrality, and closeness centrality) with 10,000 iterations were performed according to the two-sample Kolmogorov–Smirnov test [75]. The co-occurrence network and topology characteristics were determined by using the R packages “vegan”, “igraph” and “Hmisc” [76], and visualization was made with Gephi version 0.9.2 [77].

### RNA sequencing and transcriptome analysis

Eukaryotic messenger RNA (mRNA) was isolated from total RNA (1 µg per sample), which had been treated with DNase I, using magnetic bead-labeled oligo(dT)s. RNA sequencing libraries were prepared and sequenced on a DNBSEQ platform with a paired-end sequencing length of 150 bp using a commercial service (BGI, Shenzhen, China). Approximately 30 Gb of sequencing data were generated for each sample. The sequencing data were filtered to remove reads containing adaptors and low-quality reads using SOAPnuke v1.4.0, a filtering software developed by BGI (Shenzhen, China). The high-quality reads were assembled using Trinity v2.0.6 [78]. Tgicl v2.0.6 [79] was used to cluster transcripts into unigenes. Unigenes were annotated using databases including the nucleotide sequence database, the nonredundant protein sequence database (NR), Kyoto Encyclopedia of Genes and Genomes (KEGG), and SwissProt using Blast v2.2.23 [80]. Blast2GO v2.5.0 [81] was used to produce GO annotations based on NR annotation. To identify coral and Symbiodiniaceae sequences, unigenes were aligned with the genome sequences of *Acropora digitifera* [82], *Acropora millepora* [83], *Stylophora pistillata* [84], and *Pocillopora damicornis* [63, 85], *Breviolum minutum* (formerly *Symbiodinium minutum*; [ref. 86]), *Symbiodinium microadriaticum* [87], *Symbiodinium kawagutii* [88], *Symbiodinium* sp. clade C Y103, and *Symbiodinium* sp. clade A Y106 [89], using BLAST with an e-value threshold of 1e-05. Symbiodiniaceae sequences were obtained after removement of the unigenes identified as coral-derived and that cannot be clearly classified. Clean reads were mapped to unigenes classified to Symbiodiniaceae using Bowtie2 v2.2.5 [90], and gene expression levels in each sample were calculated using RSEM v1.2.8 [91]. For coral sequences, the clean reads were mapped to reference *P. damicornis* genome sequences [63] using HISAT2 v2.1.0/Bowtie2 v2.2.5 [92], and gene expression levels were calculated using RSEM v1.2.8. To compare the gene expression profiles of corals and Symbiodiniaceae, a Bray‒Curtis distance matrix was produced from the gene expression matrix (in fragments per kilobase of transcript per million mapped reads), and a principal co-ordinate analysis (PCoA) profile was generated. Differences in the transcriptomes of corals and Symbiodiniaceae among temperature treatments were tested using PERMANOVA with 999 permutations.

Based on the expression results, differentially expressed genes between groups were determined using DEseq2 [93] with a negative binomial distribution. Genes were considered significantly differentially expressed when the adjusted *P*-value was < 0.05. GO functional enrichments of differentially expressed genes (DEGs) were determined using clusterProfiler v4.0 in R [94], and *P*-values were corrected using the false discovery rate. DEG functions were considered significantly enriched when corrected *P*-values were < 0.05.

### Electronic supplementary material

Below is the link to the electronic supplementary material.

Supplementary Material 1: Module connectors in the three coral-associated bacterial networks

Supplementary Material 2: Characteristics of strain SCSIO 13291

Supplementary Material 3: Methods used in genome sequencing and processing, phylogenetic and phenotypic analyses, and vitamin assays

Supplementary Material 4: Supplementary Figures S1–S12 and Tables S1 and S3

## Data Availability

The genome of strain SCSIO 13291 was deposited under BioProject accession number PRJNA917792. The amplicon data are deposited under BioProject accession number PRJNA917712. The transcriptome data are deposited under BioProject accession number PRJNA917790. The other data are available in the main text or the supplementary materials.
