# Supplementary material for: A coral-associated actinobacterium mitigates coral bleaching under heat stress
Source: Environ Microbiome. 2023 Nov 23;18:83. doi: 10.1186/s40793-023-00540-7 (PMC10668361; doi:10.1186/s40793-023-00540-7)
Supplement: Supplementary file 2 — Supplementary Material 2: Characteristics of strain SCSIO 13291 [file 40793_2023_540_MOESM2_ESM.pdf]

Data S1 Characteristics of strain SCSIO 13291.

Colonies of strain SCSIO 13291 were Gram-stain-positive, non-motile, and facultative anaerobic. The cells were spherical or short rod-shaped and were mostly paired or clustered under aerobic culture conditions; the cells were short rod-shaped and were single or paired under anaerobic culture conditions (Fig. 1). Cells had catalase activity, while H_2S production, nitrate reduction, hydrolysis of starch and gelatin, and oxidase and urease activity were negative. The range of temperatures tested indicated that SCSIO 13291 grew on marine agar from 15-40°C (optimum, 30°C). Growth occurred at pH 6–10 (optimum, pH 8) and tolerated a range of NaCl concentrations from 0–10% (optimum, 1%). Strain SCSIO 13291 has alkaline phosphatase, esterase (C4), esterase lipase (C8), leucine arylamidase, acid phosphatase, naphthol-AS-BI-phosphohydrotase, α-galactosidase, β-galactosidase, β-glucuronidase, α-glucosidase, β-glucosidase, and N-acetyl-β-glucosaminidase but not lipase (C14), valine arylamidase, cystine arylamidase, trypsin, α-chymotrypsin, α-mannosidase, and *β*-fucosidase under aerobic conditions. Different from the results under aerobic conditions, the cells were negative for alkaline phosphatase, esterase (C4), esterase lipase (C8), leucine arylamidase, α-galactosidase, β-glucuronidase, and N-acetyl-β-glucosaminidase under anaerobic culture conditions.

The complete genome size of strain SCSIO 13291 is 3,829,285 bp. The genome was deposited under BioProject accession number PRJNA917792. The G+C content is 71.19%. A total of 3511 genes were predicted, and there are one prophage, five CRISPR and six rRNA genes (5S rRNA, 16S rRNA, and 23S rRNA are two copies).

The complete Embden-Meyerhof pathway and citrate cycle (TCA cycle) were identified in the SCSIO 13291 genome (Table 1). Genes required for serine, threonine, cysteine, methionine, arginine, proline, and histidine biosynthesis were found in SCSIO 13291. The activities of α-galactosidase, α-glucosidase, and N-acetyl-β-glucosaminidase were confirmed on the basis of both genetic and biochemical evidence. Although strains SCSIO 13291 and *P. soli* YIM S02567^T belong to the same species, there are obvious differences in the metabolic pathways between them (Table 1) and the physiological and biochemical characteristics, such as the lower NaCl tolerance and the capability to hyrolyze starch and gelatin of YIM S02567^T [1]. In addition, we noticed that strain SCSIO 13291 possesses complete sets of genes encoding heme biosynthesis, which was further proven by the heme assay (with the production of 8.46 ± 1.43 μg g^{-1} wet weight), while strain YIM S02567^T lacks such capability. In addition to the production of heme, the coral-associated bacterial strain SCSIO 13291 possesses more secondary metabolite synthesis gene clusters than strain YIM S02567^T isolated from forest soil. Five secondary metabolism regions in strain SCSIO 13291 were two terpene clusters, one non-ribosomal peptide synthetase cluster, one Class III lanthipeptide cluster like labyrinthopeptin, and one Class I lanthipeptide cluster like nisin, while only three regions were detected in *P. soli* YIM S02567^T including two terpene clusters and one Class III lanthipeptide cluster like labyrinthopeptin.

Fig. 1. Transmission electron micrographs of strain SCSIO 13291 grown on trypticase soy agar for 2 days at 30 °C under aerobic (left) and anaerobic (right) condition.

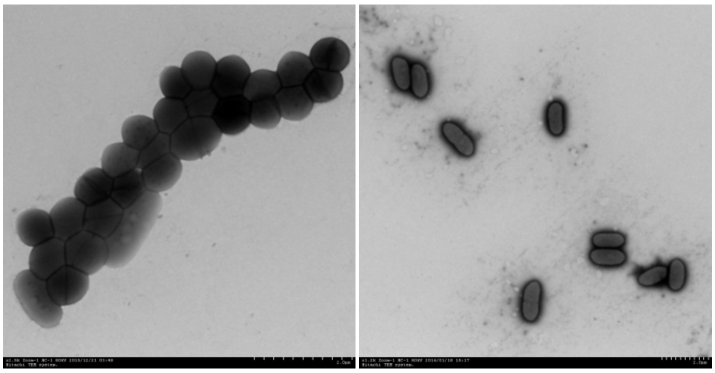

Table 1. The differences in the KEGG metabolic pathways of the genome of strains SCSIO 13291 and *Propioniciclava soli* YIM S02567^{T}.

Strains: 1, SCSIO 13291; 2, *Propioniciclava soli* YIM S02567^{T}. "+", complete KEGG metabolic pathways; "-", incomplete or nonexistent KEGG metabolic pathways.

Complete KEGG metabolic pathways	1	*2*
**Carbohydrate metabolism**		
**Central carbohydrate metabolism**		
M00001 Glycolysis (Embden-Meyerhof pathway), glucose => pyruvate (13)	+	+
M00002 Glycolysis, core module involving three-carbon compounds (7)	+	+
M00307 Pyruvate oxidation, pyruvate => acetyl-CoA(6)	+	+
M00009 Citrate cycle (TCA cycle, Krebs cycle) (16)	+	+
M00010Citrate cycle, first carbon oxidation, oxaloacetate => 2-oxoglutarate(3)	+	+
M00011Citrate cycle, second carbon oxidation, 2-oxoglutarate => oxaloacetate(13)	+	+
M00004Pentose phosphate pathway (Pentose phosphate cycle)(11)	+	+
M00006 Pentose phosphate pathway, oxidative phase, glucose 6P => ribulose 5P (5)	+	+
M00007 Pentose phosphate pathway, non-oxidative phase, fructose 6P => ribose 5P(4)	+	+
M00005 PRPP biosynthesis, ribose 5P => PRPP (1)	+	+
**Other carbohydrate metabolism**		
M00061 D-Glucuronate degradation, D-glucuronate => pyruvate + D-glyceraldehyde 3P (5)	+	+
M00854 Glycogen biosynthesis, glucose-1P => glycogen/starch (4)	+	-

M00855 Glycogen degradation, glycogen => glucose-6P (5)	+	+
M00549 Nucleotide sugar biosynthesis, glucose => UDP-glucose (4)	+	+
M00909 UDP-N-acetyl-D-glucosamine biosynthesis, prokaryotes, glucose => UDP-GlcNAc(6)	+	-
**Energy metabolism**		
**Carbon fixation**		
M00579 Phosphate acetyltransferase-acetate kinase pathway, acetyl-CoA => acetate(2)	+	+
**ATP synthesis**		
M00144 NADH:quinone oxidoreductase, prokaryotes (14)	+	+
M00151 Cytochrome bc1 complex respiratory unit (3)	+	+
M00155 Cytochrome c oxidase, prokaryotes (3)	+	+
M00157 F-type ATPase, prokaryotes and chloroplasts (8)	+	+
**Lipid metabolism**		
**Fatty acid metabolism**		
M00086 beta-Oxidation, acyl-CoA synthesis (1)	+	+
**Nucleotide metabolism**		
**Purine metabolism**		
M00048 Inosine monophosphate biosynthesis, PRPP + glutamine =>IMP (10)	+	-
M00049 Adenine ribonucleotide biosynthesis, IMP => ADP,ATP (4)	+	+
M00050 Guanine ribonucleotide biosynthesis, IMP => GDP,GTP (4)	+	+
**Pyrimidine metabolism**		
M00052 Pyrimidine ribonucleotide biosynthesis, UMP => UDP/UTP,CDP/CTP (3)	+	+
M00053 Pyrimidine deoxyribonuleotide biosynthesis, CDP => dCTP (3)	+	+
M00938 Pyrimidine deoxyribonuleotide biosynthesis, UDP => dTTP (6)	+	+
**Amino acid metabolism**		
**Serine and threonine metabolism**		
M00020 Serine biosynthesis, glycerate-3P => serine (4)	+	+
M00018 Threonine biosynthesis, aspartate => homoserine => threonine (5)	+	-
**Cysteine and methionine metabolism**		
M00021 Cysteine biosynthesis, serine => cysteine (2)	+	+

M00017 Methionine biosynthesis, apartate => homoserine => methionine (8)	+	-
**Branched-chain amino acid metabolism**		
M00019 Valine/isoleucine biosynthesis, pyruvate => valine / 2-oxobutanoate => isoleucine (5)	+	+
M00570 Isoleucine biosynthesis, threonine => 2-oxobutanoate => isoleucine (6)	+	+
M00432 Leucine biosynthesis, 2-oxoisovalerate => 2-oxoisocaproate (4)	+	+
**Lysine metabolism**		
M00016 Lysine biosynthesis, succinyl-DAP pathway, aspartate => lysine (9)	-	+
**Arginine and proline metabolism**		
M00028 Ornithine biosynthesis, glutamate => ornithine (5)	+	+
M00844 Arginine biosynthesis, ornithine => arginine (3)	+	+
M00015 Proline biosynthesis, glutamate => proline (3)	+	+
**Histidine metabolism**		
M00026 Histidine biosynthesis, PRPP => histidine (10)	+	+
**Aromatic amino acid metabolism**		
M00022 Shikimate pathway, phosphoenolpyruvate + erythrose-4P => chorismate (7)	+	+
M00024 Phenylalanine biosynthesis, chorismate => phenylpyruvate => phenylalanine (3)	+	+
M00025 Tyrosine biosynthesis, chorismate => HPP => tyrosine (3)	+	+
**Metabolism of cofactors and vitamins**		
**Cofactor and vitamin metabolism**		
M00899 Thiamine salvage pathway, HMP/HET => TMP (3)	+	-
M00120 Coenzyme A biosynthesis, pantothenate => CoA (4)	+	+
M00881 Lipoic acid biosynthesis, plants and bacteria, octanoyl-ACP => dihydrolipoyl-E2/H (2)	+	+
M00140 C1-unit interconversion, prokaryotes (3)	+	+
M00121 Heme biosynthesis, plants and bacteria, glutamate => heme (10)	+	-
M00926 Heme biosynthesis, bacteria, glutamyl-tRNA => coproporphyrin III => heme (9)	+	-
M00116 Menaquinone biosynthesis, chorismate (+ polyprenyl-PP) => menaquinol (9)	+	-
**Biosynthesis of terpenoids and polyketides**		
**Polyketide sugar unit biosynthesis**		
M00793 dTDP-L-rhamnose biosynthesis (4)	+	+

References

1. Li LL, Zhao JY, Geng Y, Zhou YG, Yuan HR, Cai M, et al. *Propioniciclava soli* sp. nov., isolated from forest soil, Yunnan, China, and reclassification of the genus *Brevilactibacter* into the genus *Propioniciclava*, and *Brevilactibacter sinopodophylli*, *Brevilactibacter flavus*, and *Brevilactibacter coleopterorum* as *Propioniciclava sinopodophylli* comb. nov., *Propioniciclava flava* comb. nov., and *Propioniciclava coleopterorum* comb. nov., respectively. Arch Microbiol. 2021;204:39.
