# Supplementary material for: A coral-associated actinobacterium mitigates coral bleaching under heat stress
Source: Environ Microbiome. 2023 Nov 23;18:83. doi: 10.1186/s40793-023-00540-7 (PMC10668361; doi:10.1186/s40793-023-00540-7)
Supplement: Supplementary file 3 — Supplementary Material 3: Methods used in genome sequencing and processing, phylogenetic and phenotypic analyses, and vitamin assays [file 40793_2023_540_MOESM3_ESM.pdf]

Data S2 Methods used in the genome sequencing and processing, phylogenetic and phenotypic analyses, and vitamin assays.

Bacterial genome sequencing and processing

Strain SCSIO 13291 was grown in trypticase soy broth (TSB; BD) at 30°C for two days. Genomic DNA was extracted using the UltraClean® microbial DNA isolation kit (Mo Bio Laboratories). The complete genome for SCSIO 13291 was sequenced on the PacBio RS platform and the Illumina HiSeq 4000 platform at the Beijing Genomics Institute (BGI, Shenzhen, China). Four SMRT cell zero-mode waveguide arrays of sequencing were used by the PacBio platform to generate the subreads set. Subreads with lengths <1kb were removed. The program Pbdagcon (https://github.com/PacificBiosciences/pbdagcon) was used for self-correction. Draft genome unitigs, which are uncontested groups of fragments, were assembled using Celera Assembler against a high-quality corrected circular consensus sequence subreads set. To improve the accuracy of the genome sequence, GATK (https://www.broadinstitute.org/gatk/) and SOAP tool packages (SOAP2, SOAPsnp, and SOAPindel) were used to make single-base corrections. The above genome was annotated with Non-Redundant Protein Database (NR), Swiss-Prot, Gene Ontology (GO), Kyoto Encyclopedia of Genes and Genomes (KEGG) and Clusters of Orthologous Groups (COG) databases. Pathway reconstruction was performed using KEGG Mapper (https://www.genome.jp/kegg/mapper.html). The genome of the strain was also analyzed using antiSMASH [1] to explore its biosynthetic potential.

Phylogeny based on the 16S rRNA gene

The full-length 16S rRNA gene sequence of strain SCSIO 13291 was extracted from the genome sequence and used for phylogenetic analysis. The 16S rRNA gene sequence was used to infer the nearest phylogenetic relatives using the EzBioCloud platform (http://eztaxon-e.ezbiocloud.net/) [2]. Sequences from strain SCSIO 13291, as well as its determined closest relatives, were aligned using ClustalW [3, 4], which allowed the reconstruction of phylogenetic tree with neighbor-joining [5], as implemented in the program MEGA X [6]. The topology of the phylogenetic tree was evaluated using bootstrap analysis based on 1000 resamplings, as described previously [7, 8].

Morphological and physiochemical characterization

Cell morphology was observed by using light microscopy (OLYMPUS BX53) and transmission electron microscopy (Hitachi TEM System HC-1) with cells grown in TSB for two days at 30°C. Growth was examined under anaerobic conditions on trypticase soy agar (TSA; BD) plates in an anaerobic jar (Mitsubishi Gas Chemical Co., Inc.) contained 1 mg L^{-1} resazurin as an oxygen indicator. Gram-staining behavior and oxidase and catalase activity were tested according to the classic microbiological methods [9]. Motility was confirmed by observing the growth spread of cells in semi-solid medium. Growth on TSA medium was assessed at different temperatures (10, 15, 20, 25, 30, 35, 40, and 45°C), at various pH values (pH 4–13, at intervals of 1 pH unit) and with different NaCl concentrations (0–15%, with an interval of 1% [w/v]) after four days of incubation. The strain was incubated on peptone-gelatin medium at 30°C for three days to determine the hydrolysis of gelatin.

Additional enzymatic features were assessed by using the API ZYM system (bioMérieux).

Vitamin analysis

Strain SCSIO 13291 was grown in TSB at 30°C for four days. Subsequently, the culture broth was centrifuged (10,000×*g*, 15 min, 4°C), and the supernatant was filtered through a 0.22 µm GSWP membrane (Merck) and then used for extracellular vitamin analysis. The content of folic acid was determined by microbiological assays following the protocols by Hugenschmidt et al. [10] with the utilization of *Lactobacillus rhamnosus* (ATCC 7469). The vitamin B3 content was determined by high-performance liquid chromatography according to a previous method [11].

References

1. Blin K, Shaw S, Kloosterman AM, Charlop-Powers Z, van Wezel GP, Medema MH, et al. antiSMASH 6.0: improving cluster detection and comparison capabilities. Nucleic Acids Res. 2021;49:W29–W35.
2. Yoon SH, Ha SM, Kwon S, Lim J, Kim Y, Seo H, et al. Introducing EzBioCloud: a taxonomically united database of 16S rRNA gene sequences and whole-genome assemblies. Int J Syst Evol Microbiol. 2017;67:1613–7.
3. Larkin MA, Blackshields G, Brown NP, Chenna R, McGettigan PA, McWilliam H, et al. Clustal W and clustal X version 2.0. Bioinformatics. 2007;23:2947–8.
4. Thompson JD, Higgins DG, Gibson TJ. Clustal-W - Improving the Sensitivity of Progressive Multiple Sequence Alignment through Sequence Weighting, Position-Specific Gap Penalties and Weight Matrix Choice. Nucleic Acids Res. 1994;22:4673–80.
5. Saitou N, Nei M. The Neighbor-Joining Method - a New Method for Reconstructing Phylogenetic Trees. Mol Biol Evol. 1987;4:406–25.
6. Kumar S, Stecher G, Li M, Knyaz C, Tamura K. MEGA X: Molecular Evolutionary Genetics Analysis across Computing Platforms. Mol Biol Evol. 2018;35:1547–9.
7. Felsenstein J. Confidence-Limits on Phylogenies - an Approach Using the Bootstrap. Evolution. 1985;39:783–91.
8. Pruesse E, Peplies J, Glöckner FO. SINA: Accurate high-throughput multiple sequence alignment of ribosomal RNA genes. Bioinformatics. 2012;28:1823–9.
9. Li Q, Zheng Y, Guo A, Chen Y, Zhang S, Li J. *Pseudokineococcus galaxeicola* sp. nov., isolated from mucus of a stony coral. Int J Syst Evol Microbiol. 2020;70:5671–5.
10. Hugenschmidt S, Schwenninger SM, Lacroix C. Concurrent high production of natural folate and vitamin B12 using a co-culture process with SM39 and DF13. Process Biochem. 2011;46:1063–70.
11. Jin P, Xia L, Li Z, Che N, Zou D, Hu X. Rapid determination of thiamine, riboflavin, niacinamide, pantothenic acid, pyridoxine, folic acid and ascorbic acid in Vitamins with Minerals Tablets by high-performance liquid chromatography with diode array detector. J Pharm Biomed Anal. 2012;70:151–7.
