# Supplementary material for: A coral-associated actinobacterium mitigates coral bleaching under heat stress
Source: Environ Microbiome. 2023 Nov 23;18:83. doi: 10.1186/s40793-023-00540-7 (PMC10668361; doi:10.1186/s40793-023-00540-7)
Supplement: Supplementary file 4 — Supplementary Material 4: Supplementary Figures S1–S12 and Tables S1 and S3 [file 40793_2023_540_MOESM4_ESM.docx]

Supplementary Materials for

**A coral-associated actinobacterium mitigates coral bleaching under heat stress**

Jie Li,* Yiyang Zou, Qiqi Li, Jian Zhang, David G. Bourne, Yuanjiao Lyu, Cong Liu, Si Zhang

*Corresponding author. Email: [lijietaren@scsio.ac.cn](mailto:lijietaren@scsio.ac.cn)

**This file includes:**

Figs. S1 to S12

Tables S1 to S3

Datasets S1 to S2

**Fig. S1. Neighbour-joining phylogenetic tree reconstructed based on 16S rRNA gene sequences, showing the position of strains SCSIO 13291 and the type strains of related taxa within the family Propionibacteriaceae.** Only bootstrap values (percentages of 1000 replications) > 50 % were shown. The strain characterized in this study is shown in bold type. All ambiguous positions were removed for each sequence pair (pairwise deletion option). Bar, 0.01 changes per nucleotide position.

**Fig. S2 Photos of the coral nubbins before inoculation and at the end of the experiment.** “Before” corresponds to the time when the temperature reached 31°C and before the inoculation that also occurred on day 9. “After” corresponds to the end of the experiment (day 39).

**Fig. S3. NMDS plots of the *Pocillopora damicornis* associated bacterial communities at T1 (day 9, A), T2 (day 29, B), and T3 (day 39, C) (n = 5).**

**Fig. S4.** **Confocal laser scanning microscopy results of coral fragments at 31°C.** (A, F) Coral fragment collected at the T1 timepoint; TAMRA/SE signal (A), Coral GFP (green), algal chlorophyll (red), and TAMRA/SE labeled bacteria (yellow) overlaid image (F). (B, G) SCSIO 13291-treated coral fragment collected at the T2 timepoint; TAMRA/SE signal (B), coral GFP (green), algal chlorophyll (red), and TAMRA/SE-labeled bacteria (yellow) overlaid image (G). (C, H) FASW-treated coral fragment collected at the T2 timepoint; TAMRA/SE signal (C), Coral GFP (green), algal chlorophyll (red), and TAMRA/SE labeled bacteria (yellow) overlaid image (H). (D, I) SCSIO 13291-treated coral fragment collected at the T3 timepoint; TAMRA/SE signal (D), coral GFP (green), algal chlorophyll (red), and TAMRA/SE-labeled bacteria (yellow) overlaid image (I). (E, J) FASW-treated coral fragment collected at the T3 timepoint; TAMRA/SE signal (E), Coral GFP (green), algal chlorophyll (red), and TAMRA/SE labeled bacteria (yellow) overlaid image (J). White arrows showed TAMRA/SE labeled cells.

**Fig. S5. The relative abundances of different phyla/classes in the three coral-associated bacterial networks.**

**Fig. S6.** **Comparison of node-level topological features between the three coral-associated bacterial networks.** Significant differences were determined using the Kolmogorov–Smirnov test. (A) Degree, (B) Betweenness centrality, (C) Closeness centrality.

**Fig. S7.** ***Zi*-*Pi* plots showing the distribution of ASVs based on their topological roles in the three networks.**

**Fig. S8.** **Principal coordinate analysis (PCoA) plot representing the differences in the overall gene expression patterns in corals at T1 (A), T2 (B), and T3 (C).**

**Fig. S9.** **Principal coordinate analysis (PCoA) plot representing the differences in the overall gene expression patterns in Symbiodiniaceae endosymbionts at T1 (A), T2 (B), and T3 (C).**

**Fig. S10.** **Differential gene expression between strain SCSIO 13291-treated and FASW-treated corals at T2 (A) and T3 (C). Gene Ontology analysis for differentially expressed genes between strain SCSIO 13291-treated and FASW-treated corals at T2 (B) and T3 (D).**

**Fig. S11.** **Differential gene expression between strain SCSIO 13291-treated and FASW-treated coral-associated endosymbiotic algae at T2 (A) and T3 (C). Gene Ontology analysis for differentially expressed genes between strain SCSIO 13291-treated and FASW-treated coral-associated endosymbiotic algae at T2 (B) and T3 (D).**

**Fig. S12.** **Differentially expressed tetratricopeptide repeat protein 28-like between strain SCSIO 13291-treated and FASW-treated corals at T2 and T3.**

**Table S1. Topological properties of the coral-associated bacterial networks.**

| Networks | 27°C | 31°C | 31°C + SCSIO 13291 |
| --- | --- | --- | --- |
| **Empirical networks** |  |  |  |
| Nodes | 227 | 313 | 284 |
| Edges | 761 | 3501 | 1173 |
| Positive links  Negative links | 761  0 | 3500  1 | 1172  1 |
| Average path length | 1.098 | 1.986 | 1.265 |
| Average degree | 6.704 | 22.370 | 8.260 |
| Graph density  Modularity (the number of modules) | 0.030  0.735 (53) | 0.071  0.420 (34) | 0.029  0.853 (51) |
| Clustering coefficient | 0.998 | 0.995 | 0.995 |
| **Random networks (SD)** |  |  |  |
| Clustering coefficient | 0.029 (0.004) | 0.071 (0.001) | 0.029 (0.002) |
| Modularity | 0.350 (0.007) | 0.168 (0.003) | 0.309 (0.006) |
| Small World coefficient | 95.682 (13.580) | 14.821 (0.329) | 78.951 (8.120) |

SD, standard deviation

**Table S2. Module connectors in the three coral-associated bacterial networks.**

EXCEL FILE

**Table S3. The results of BIOLOG GEN III MicroPlate assays of strain SCSIO 13291 labeled and unlabeled with TAMRA/SE.**

| **Cell no.** | **index** | **SCSIO 13291** | **SCSIO 13291 stained with TAMRA/SE** |
| --- | --- | --- | --- |
| A1 | Negative Control | - | - |
| A2 | Dextrin | + | + |
| A3 | D-Maltose | - | - |
| A4 | D-Trehalose | - | - |
| A5 | D-Cellobiose | - | - |
| A6 | Gentiobiose | + | + |
| A7 | Sucrose | + | + |
| A8 | D-Turanose | + | + |
| A9 | Stachyose | + | + |
| A10 | Positive Control | + | + |
| A11 | pH 6 | + | + |
| A12 | pH 5 | - | - |
| B1 | D-Raffinose | + | + |
| B2 | α-D-Lactose | + | + |
| B3 | D-Melibiose | + | + |
| B4 | β-Methyl-D-Glucoside | - | - |
| B5 | D-Salicin | + | + |
| B6 | N-Acetyl-D-Glucosamine | - | - |
| B7 | N-Acetyl-β-D-Mannosamine | - | - |
| B8 | N-Acetyl-D-Galactosamine | - | - |
| B9 | N-Acetyl Neuraminic Acid | + | + |
| B10 | 1% NaCl | + | + |
| B11 | 4% NaCl | + | + |
| B12 | 8% NaCl | + | + |
| C1 | α-D-Glucose | + | + |
| C2 | D-Mannose | - | - |
| C3 | D-Fructose | - | - |
| C4 | D-Galactose | - | - |
| C5 | 3-Methyl Glucose | + | + |
| C6 | D-Fucose | + | + |
| C7 | L-Fucose | + | + |
| C8 | L-Rhamnose | + | + |
| C9 | Inosine | - | - |
| C10 | 1% Sodium Lactate | - | - |
| C11 | Fusidic Acid | - | - |
| C12 | D-Serine | - | - |
| D1 | D-Sorbitol | + | + |
| D2 | D-Mannitol | + | + |
| D3 | D-Arabitol | - | - |
| D4 | myo-Inositol | - | - |
| D5 | Glycerol | + | + |
| D6 | D-Glucose-6-PO_4_ | + | + |
| D7 | D-Fructose-6-PO_4_ | + | + |
| D8 | D-Aspartic Acid | + | + |
| D9 | D-Serine | - | - |
| D10 | Troleandomycin | - | - |
| D11 | Rifamycin SV | - | - |
| D12 | Minocycline | - | - |
| E1 | Gelatin | - | - |
| E2 | Glycyl-L-Prolin | - | - |
| E3 | L-Alanine | + | + |
| E4 | L-Arginine | - | - |
| E5 | L-Aspartic Acid | + | + |
| E6 | L-Glutamic Acid | + | + |
| E7 | L-Histidine | + | + |
| E8 | L-Pyroglutamic Acid | + | + |
| E9 | L-Serine | - | - |
| E10 | Lincomycin | - | - |
| E11 | Guanidine HCl | - | - |
| E12 | Niaproof 4 | - | - |
| F1 | Pectin | + | + |
| F2 | D-Galacturonic Acid | + | + |
| F3 | L-Galactonic Acid Lactone | + | + |
| F4 | D-Gluconic Acid | + | + |
| F5 | D-Glucuronic Acid | + | + |
| F6 | Glucuronamide | + | + |
| F7 | Mucic Acid | - | - |
| F8 | Quinic Acid | - | - |
| F9 | D-Saccharic Acid | - | - |
| F10 | Vancomycin | - | - |
| F11 | Tetrazolium Violet | - | - |
| F12 | Tetrazolium Blue | - | - |
| G1 | p-Hydroxy-Phenylacetic Acid | - | - |
| G2 | Methyl Pyruvate | - | - |
| G3 | D-Lactic Acid Methyl Ester | - | - |
| G4 | L-Lactic Acid | - | - |
| G5 | Citric Acid | - | - |
| G6 | α-Keto-Glutaric Acid | - | - |
| G7 | D-Malic Acid | - | - |
| G8 | L-Malic Acid | - | - |
| G9 | Bromo-Succinic Acid | - | - |
| G10 | Nalidixic Acid | + | + |
| G11 | Lithium Chloride | + | + |
| G12 | Potassium Tellurite | + | + |
| H1 | Tween 40 | - | - |
| H2 | γ-Amino-Butryric Acid | - | - |
| H3 | α-Hydroxy-Butyric Acid | - | - |
| H4 | β-Hydroxy-D,L Butyric Acid | - | - |
| H5 | α-Keto-Butyric Acid | - | - |
| H6 | Acetoacetic Acid | + | + |
| H7 | Propionic Acid | - | - |
| H8 | Acetic Acid | + | + |
| H9 | Formic Acid | - | - |
| H10 | Aztreonam | + | + |
| H11 | Sodium Butyrate | - | - |
| H12 | Sodium Bromate | - | - |

**Dataset S1. (separate file)**

**Characteristics of strain SCSIO 13291**.

**Dataset S2. (separate file)**

**Methods used in genome sequencing and processing, phylogenetic and phenotypic analyses, and vitamin assays.**
